# Identification of genetic loci associated with crude protein and mineral concentrations in alfalfa (*Medicago sativa*) using association mapping

**DOI:** 10.1186/s12870-017-1047-x

**Published:** 2017-06-06

**Authors:** Congjun Jia, Xinming Wu, Min Chen, Yunqi Wang, Xiqiang Liu, Pan Gong, Qingfang Xu, Xuemin Wang, Hongwen Gao, Zan Wang

**Affiliations:** 1 Institute of Animal Sciences, Chinese Academy of Agricultural Sciences, Beijing, 100193 China; 2 Institute of Animal Husbandry and Veterinary Science, Shanxi Academy of Agricultural Sciences, Taiyuan, 030032 China; 3 College of Animal Science and Veterinary Medicine, Shanxi Agricultural University, Taigu, 030801 China

**Keywords:** Alfalfa, Association mapping, Crude protein, Mineral elements, Simple sequence repeat (SSR)

## Abstract

**Background:**

Alfalfa (*Medicago sativa*) is one of the most important legume forage species in China and many other countries of the world. It provides a quality source of proteins and minerals to animals. Genetic underpinnings for these important traits, however, are elusive. An alfalfa (*M. sativa*) association mapping study for six traits, namely crude protein (CP), rumen undegraded protein (RUP), and four mineral elements (Ca, K, Mg and P), was conducted in three consecutive years using a large collection encompassing 336 genotypes genotyped with 85 simple sequence repeat (SSR) markers.

**Results:**

All the traits were significantly influenced by genotype, environment, and genotype × environment interaction. Eight-five significant associations (*P* < 0.005) were identified. Among these, five associations with Ca were repeatedly observed and six co-localized associations were identified.

**Conclusions:**

The identified marker alleles significantly associated with the traits provided important information for understanding genetic controls of alfalfa quality. The markers could be used in assisting selection for the individual traits in breeding populations for developing new alfalfa cultivars.

**Electronic supplementary material:**

The online version of this article (doi:10.1186/s12870-017-1047-x) contains supplementary material, which is available to authorized users.

## Background

Alfalfa (*Medicago sativa*) has long been cultivated as the most important legume forage crop in the world. The widespread use of alfalfa is due to its high forage quality. Alfalfa forage is high in crude protein (CP), but its rumen undegraded protein (RUP) content is low. Most proteins of alfalfa are degraded in rumen into small N-containing molecules, instead of essential amino acids that are required by animals, subsequently decreasing the utilization value of alfalfa N and consequently releasing to the environment and causing pollutions [1, 2]. K, Ca, Mg, and P are essential minerals to animals, which are involved in various physiological activities regulating metabolisms [3], affecting growth and development, and improving the quality of animal products. For example, an increased concentration of K in diet could reduce trans-fatty acids in milk [4], prevent metabolic alkalosis and decrease the risk of hypocalcemia and milk fever in cows [5]. Thus, identification of genes that control aforementioned forage quality component traits will provide insights for alfalfa breeding programs.

Most quality traits, like CP, RUP, and mineral elements, are quantitative traits coded by multiple genes, influenced by environment, and complexed by genotype by environment interaction. Traditional linkage mapping is the most common approach to detect quantitative trait loci (QTLs) conditioning complex traits in plants. To date, several QTL studies in mineral elements have been reported based on linkage mapping investigations in *Oryza sativa* [6, 7], *Triticum aestivum* [8, 9], and *Brassica napus* [10], and *Zea mays* [11, 12]. Compared with linkage analysis, association mapping is a recently-emerged alternative robust tool to overcome the restriction of classical QTL mapping [13]. It has been widely applied in the major crop species to detect QTLs by establishing marker-trait associations [14–18]. Recently, Huang et al. [19], and Nawaz et al. [20], respectively reported marker-trait associations (MTAs) for mineral elements in rice using association mapping. But for alfalfa, little information is available for the genetic structures in CP, RUP and mineral elements, by linkage analysis or association mapping. In the present study, genome wide association (GWAS) approach was used to investigate SSR markers associated with the six quality traits in a core collection of alfalfa.

## Results

### Phenotypic variations in an alfalfa association mapping population

**Fig. 1:**
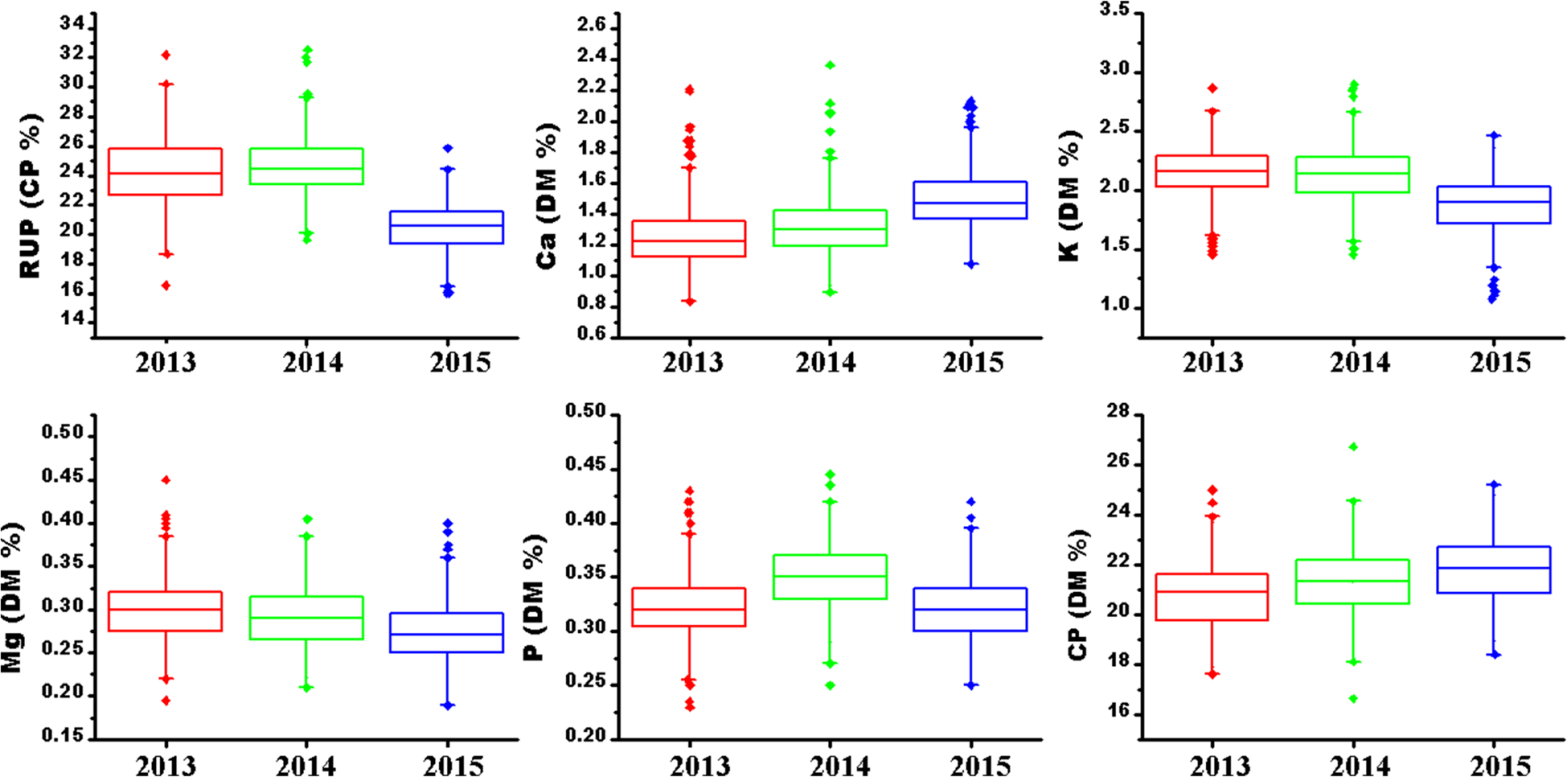
Boxplot of phenotypic distribution for six traits in 3 years

The six measured traits for 336 alfalfa genotypes are given in Table 1. The average values of crude protein (CP) and Ca were slightly increased from 2013 to 2015, while K and Mg were just the opposite (Table 1; Fig. 1). All the datasets showed normal or nearly normal distributions (Table 1). The ANOVA results indicated that all the traits were significantly influenced by genotype, environment and genotype × environment interaction (*P* < 0.001) (Table 1). For genotype, the most notable effect was detected in Ca (H = 0.79) and the smallest in RUP (0.56) (Table 1). The most significant environment and genotype × environment effects were both observed in rumen undegraded protein (RUP), while the slightest in Mg and K (Table 1). The results indicated sufficient genetic variability existed in the alfalfa collection that was appropriate for the association mapping research. Interestingly, Pearson’s phenotypic correlation coefficients were highly significant between the traits (*P* < 0.001) (Table 2). Among 15 pairs of the six traits, eight pairs showed positive correlations while the remaining seven pairs appeared to be negatively correlated (Table 2).

**Table 1:** Phenotypic variation for six quality traits in alfalfa in 3 years

Traits	Year	Min	Max	Mean ± SD	Skewness	Kurtosis	*H*	Genotype	Year	Genotype ×year
CP (DM%)	2013	17.62	25.01	20.77 ± 1.33	−0.06	−0.13	0.67	10.14***	266.06***	3.29***
	2014	16.66	26.73	21.29 ± 1.40	−0.01	0.32				
	2015	18.39	25.21	21.85 ± 1.37	0.01	-0.45				
RUP (CP%)	2013	16.56	32.18	24.28 ± 2.35	0.06	0.11	0.56	8.51***	2176.46***	3.49***
	2014	19.65	32.52	24.63 ± 1.87	0.50	1.57				
	2015	16.05	25.88	20.54 ± 1.60	−0.01	0.10				
Ca (DM%)	2013	0.84	2.21	1.26 ± 0.20	1.36	3.62	0.79	13.79***	916.8***	2.87***
	2014	0.90	2.37	1.32 ± 0.90	1.13	3.79				
	2015	1.08	2.14	1.51 ± 0.19	0.82	0.74				
K (DM%)	2013	1.45	2.87	2.15 ± 0.23	−0.20	0.45	0.74	6.18***	436.11***	1.6***
	2014	1.46	2.90	2.14 ± 0.25	0.09	0.24				
	2015	1.08	2.47	1.88 ± 0.24	−0.45	0.49				
Mg (DM%)	2013	0.20	0.45	0.30 ± 0.04	0.60	0.88	0.76	10.15***	257.46***	2.53***
	2014	0.21	0.41	0.29 ± 0.03	0.33	0.27				
	2015	0.19	0.40	0.27 ± 0.04	0.68	0.86				
P (DM%)	2013	0.23	0.43	0.32 ± 0.03	0.35	1.20	0.59	5.82***	401.95***	2.41***
	2014	0.25	0.45	0.35 ± 0.03	0.10	0.20				
	2015	0.25	0.42	0.32 ± 0.03	0.32	0.17				

H, Broad-sense heritability

***indicate Significance at *P* < 0.001 levels

**Table 2:** Correlation analysis for six forage quality related traits in alfalfa

	CP	Ca	K	Mg	P
Ca	0.178^***^				
K	0.163^***^	−0.622^***^			
Mg	0.197^***^	0.704^***^	−0.525^***^		
P	0.497^***^	−0.399^***^	0.595^***^	-0.234^***^	
RUP	-0.368^***^	−0.558^***^	0.376^***^	−0.285^***^	0.212^***^

*** indicate Significance at *P* < 0.001

### Association analysis

**Fig. 2:**
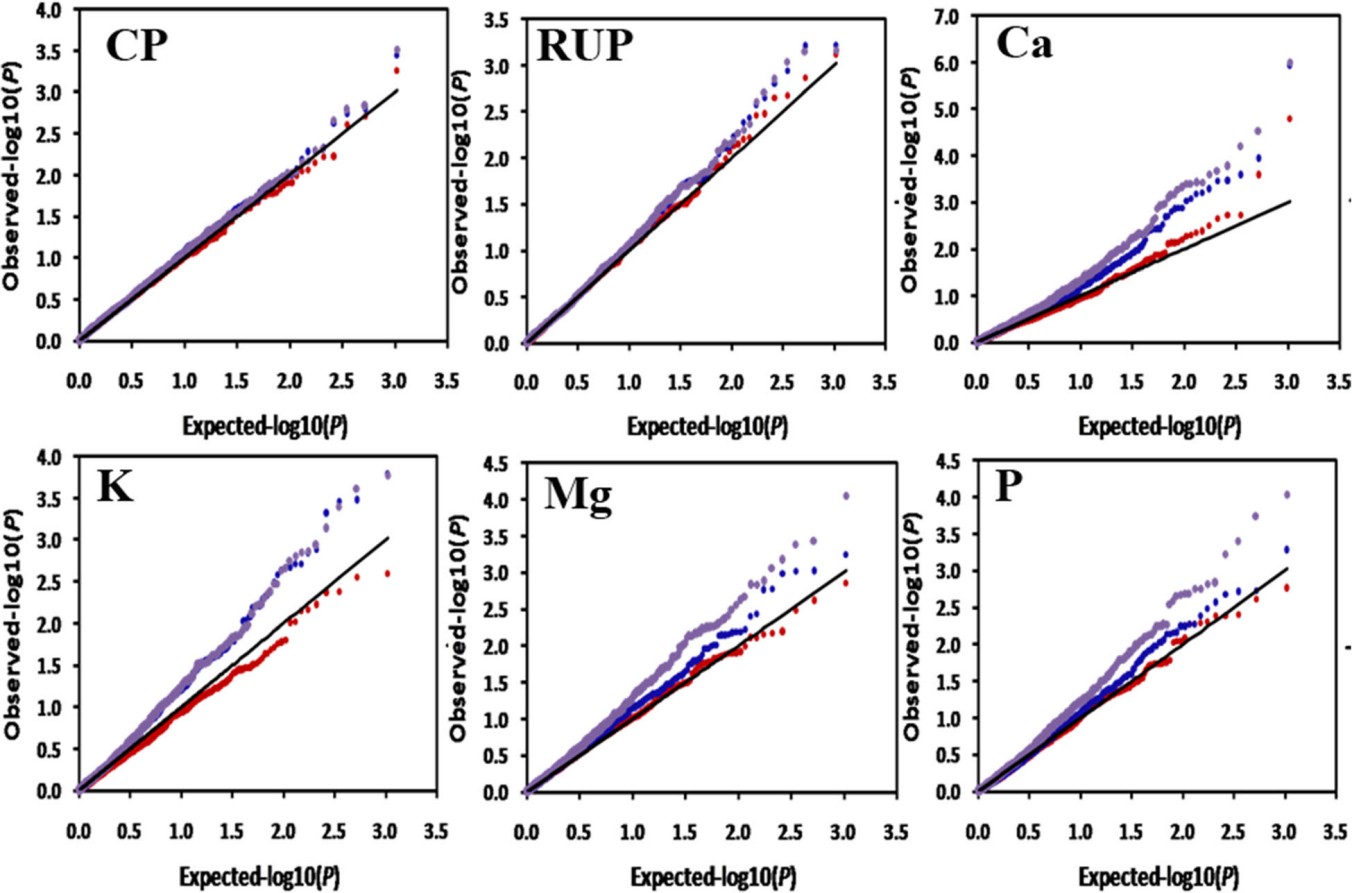
QQ plot of observed versus expected *P* – values using three different models for six traits. The GLM Naive (*violet trace*), GLM Q-model (*blue trace*) and MLM (*red trace*)

To control false positive trait-marker associations, three models, namely general linear model (GLM), Q, and Q + K, were compared with each other using the quantile-quantile (Q-Q) plot shown in Fig. 2. In general, the *P* value obtained from the Q + K model was more close to the expected *P* value than the other two models (Fig. 2). Using the respective mean phenotyping values of the 3 years, 256, 192, and 85 marker-trait associations (MTAs) were detected by GLM, Q, and Q + K model, respectively (Table 3). The result showed that the false positives were appropriately controlled by using the Q + K model. Therefore, the following analyses were based on the Q + K model.

**Table 3:** Association summary for six measured traits in alfalfa using three models in 3 years

Traits	Year	GLM		Q		Q + K	
		No of alleles	R^2^ (%)	No of alleles	R^2^ (%)	No of alleles	R^2^ (%)
CP	2013	5	2.13–4.09	5	2.13–4.01	3	2.89–3.81
	2014	3	2.35–4.02	2	2.21–3.75	2	3.25–3.58
	2015	8	2.10–3.87	10	2.31–3.84	8	2.88–3.54
RUP	2013	8	2.15–3.51	8	2.11–3.59	6	2.69–3.69
	2014	5	2.42–4.10	4	2.16–4.47	4	2.88–4.80
	2015	3	2.13–3.32	3	2.20–3.23	2	2.83–3.03
Ca	2013	29	2.06–7.40	24	2.26–6.92	8	2.47–5.99
	2014	20	2.16–9.65	13	2.09–8.95	6	3.15–8.20
	2015	30	2.13–6.10	19	1.94–6.67	10	2.44–6.95
K	2013	15	2.31–4.76	16	2.08–4.80	4	3.54–3.86
	2014	11	2.19–3.60	11	2.15–3.80	0	
	2015	17	2.16–7.09	14	2.15–5.03	2	2.59–3.79
Mg	2013	15	2.09–5.18	8	1.94–3.42	3	2.65–3.13
	2014	11	2.19–3.78	6	2.16–3.31	0	
	2015	26	2.24–6.46	18	2.04–5.23	9	2.58–5.27
P	2013	14	2.20–4.76	7	2.24–3.49	6	2.43–2.98
	2014	13	2.15–4.50	12	2.05–5.42	5	3.00–3.59
	2015	23	2.16–7.41	12	2.12–5.97	7	2.64–3.69

R^2^, the explained phenotypic variance

Based on the Q + K model, a total of 85 MTAs, respectively, 30 in 2013, 17 in 2014 and 38 in 2015, were detected significantly for the six measured traits with 46 SSR markers in at least 1 year (Additional file 1). For CP, three, two and eight MTAs were significant in 3 years, respectively, with the explained phenotypic variance (R^2^) ranging from 2.88 to 3.81% (Additional file 1). For RUP, six, four, and two MTAs were identified to be significant in 3 years, respectively, with the R^2^ from 2.69 to 4.80%. For Ca, eight, six, and ten significant MTAs were detected in 3 years, respectively, with the R^2^ from 2.44 to 8.20%. For K, four, zero, and two MTAs were significant in 3 years, respectively, with the R^2^ from 2.59 to 3.86%. For Mg, three, zero, and nine MTAs were detected in 3 years, respectively, with the R^2^ from 2.58 to 5.27%. For P, six, five, and seven MTAs were detected in 3 years, respectively, with the R^2^ from 2.43 to 3.69%.

Among the 85 MTAs, most were detected only in 1 year for CP, RUP, K, Mg, and P except those for Ca. Five MTAs with Ca were repeatedly observed in 2 years. The alleles, m215_182 and m583_128, which located in chromosome one, were respectively detected in 2013 and 2015, and 2013 and 2014. The alleles, m13_170 and m13_173, which located in chromosome two, were detected in 2013 and 2015, 2014 and 2015, respectively. The allele m429_245 located in chromosome four was detected in 2014 and 2015 (Additional file 1).

Among these associated alleles, some alleles were found associated with multiple traits especially in 2015. Five alleles, m13_170, m19_128, m350_342, m429_245 and m53_151, co-associated with Ca and Mg in 2015, and the other three alleles m19_162, m359_175 and m46_123 co-associated with CP and P in 2015 (Additional file 1). One allele, m630_301 was co-associated with K and Mg in 2013, and m225_203 co-associated with Ca and P in 2014. The significant associations of the same alleles with multiple traits might be the result of pleiotropy.

### Mining of elite alleles

The phenotypic effect of each allele that significantly associated with each of the six traits was shown in Additional file 1. In this study, the alleles with positive effects are considered to be elite alleles for all the six measured traits. Among the 13 alleles associated with CP, 11 showed positive effects. The allele m257_203 had the most positive phenotypic effect (3.16) in 2015. For RUP, five alleles had positive effects and allele m83_155 had the most positive phenotypic effect (3.59) in 2013. Only one allele m520_148 showed a positive effect (0.51) associated with K. The allele m630_301 had the most positive phenotypic effect (0.09) in 2013 among the 12 alleles associated with Mg. The allele m225_203 had the most positive phenotypic effect (0.09) associated with P in 2004. However almost all of elite alleles mentioned above were only associated in 1 year. The m13_170 was only one that stably associated with Ca over different years although it has small positive effect (0.06).

## Discussion

In this study, alfalfa genotypes showed significant levels of genetic diversity (*P* < 0.001), as revealed by ANOVA of all the six traits, and the most traits were highly heritable, showing a broad variation among the alfalfa genotypes (Table 1). All the traits were significantly influenced by environment and genotype × environment interactions. Two important environment factors, precipitation and temperature, were investigated during the 3 years (Additional file 2). There was no any significant difference on the temperature during the 3 years. Whereas, some significant difference was observed for precipitation especially in April and May among 3 years which was the important development stage of the alfalfa. Obviously, the precipitation is the main factor leading to environmental variance and ultimately affect the six quality traits in the study. Correlation analysis of different traits is considered very useful in exploring interrelationships. In the present study, numerous significant correlations were observed between the six different traits (Table 1). These correlations may be due to the impact of a single gene on multiple traits or co-association of physically closely located genes [20].

Association mapping is a reliable method for quickly identifying the loci responsible for natural variants in a target phenotype [21]. Recently, association mapping was also used to identify loci associated with the biomass yield and stem composition [22], and verticillium wilt resistance [23] in tetraploid alfalfa and forage yield and nutritive value in diploid alfalfa [24]. However, less information is known about the association of SSR loci with crude protein, RUP, and mineral elements in plant species. Huang et al. [19] reported a total of 20 marker-trait associations identified for the five mineral elements in rice. Nawaz et al. [20] identified 60 marker loci associated with eight grain elemental concentrations in brown rice. The present study is the first attempt in characterizing the alfalfa genotypes using genomic SSR markers for crude protein, RUP, and four mineral elements in a diverse set of worldwide collection of alfalfa accessions. A total of 85 MTAs was identified as associated with measured traits based on the association analysis using the MLM model (Additional file 1). Most of the loci associated with the six traits were identified only in a specific year, suggesting their expression for these traits is significantly influenced by the environment. However, stable associations with Ca were identified in our study, such as the alleles m215_182 and m583_128 located in chromosome one, m13_170 and m13_173 located in chromosome two, m429_245 in chromosome four (Additional file 1). Therefore, these associated markers and identified genotypes with favorable alleles could be used for marker assisted selection in alfalfa breeding after validation.

It is reported that co-localized or pleiotropic associations may be helpful to reveal some important genomic regions or genes for the desirable traits [19]. In this study, several co-localized associations were detected. For example, m13_170 was found associated with Ca, and Mg in Chromosome two, suggesting that it may be possible to select for high Ca, and Mg lines using molecular markers in these regions (Additional file 1). m19_162 was found associated with crude protein and P in Chromosome two, indicating that it may be possible to select for high crude protein and P lines (Additional file 1). Similar results were reported in rice [19], *Aegilops tauschii* [25], and *Festuca arundinacea* [26]. Furthermore, the markers associated with more than one trait may be effectively used of improving more than one trait in marker assisted selection. The phenomenon of co-localization may be caused by pleiotropy of the same gene involved in the metabolism and physiological processes of several elements or is the presence of clustered genes that are tightly linked responsible for the accumulation of different elements in rice grain [27].

Because of the complex nature of the studied traits and limited markers used, most of the experimental results showed poor repeatability and lower explained phenotypic variance (<10%) as indicated by the similar research [14, 18, 25, 26] . But some of elite alleles were still detected associated with the studies traits. These will be useful for molecular marker assist selection breeding in alfalfa compared to traditional phenotype-based selection.

## Conclusions

The present study is the first attempt in characterizing the alfalfa genotypes using genomic SSR markers for CP, RUP, and four mineral elements (Ca, K, Mg and P) traits in a diverse set of worldwide collection of alfalfa accessions. Our results showed that this alfalfa association panel could be appropriate for association analyses targeting complex agronomics traits with optimal association models. The markers associated to the QTLs in the study could be effectively used in improving locally well adapted germplasm by marker assisted introgression of desirable alleles.

## Methods

### Plant materials and experimental design

The association panel was consisted of a total of 336 individual genotypes from 75 cultivated tetraploid alfalfa accessions (Additional file 3) [28]. Nine Chinese accessions were obtained from the National Herbage Germplasm Bank of China; two accessions from Syria, one from Libya and one from Sudan provided by the Institute of Animal Science, Chinese Academy of Agricultural Science (Beijing, China).The other 62 accessions were provided by the USDA National Plant Germplasm System (NPGS). The field experiments were performed on the experimental station of the Institute of Dry Farming, Hebei Academy of Agriculture and Forestry Sciences in Hengshui, Hebei province, established in May 2012 (37°44′N; 115°42′E). The mean annual precipitation of 484 mm (65% in July and August), evaporation of 1670 mm, and average temperature of 13.2 °C, sunshine duration of 2546 h, relative humidity of 63%, frost-free period of 206 days in long-term. Soil type in this area is typically silty loam, with PH of 7.88, salt of 0.053%, organic matter of 16.5 g kg^−1^, alkali–hydrolysable N of 60.4 mg kg^−1^, available P of 12.5 mg kg^−1^, available K of 201.7 mg kg^−1^ within the top 20 cm soil. The experimental design was reported previously [28].

### Phenotyping

The analyses for the contents of crude protein (CP), rumen un-degraded protein (RUP), and mineral elements (Ca, K, Mg and P) were performed on all plant samples for 3 years (2013, 2014 and 2015). The biomass above the ground was harvested at the early flowering stage and dried at 60 °C for 48 h. Then plants were ground to pass a 1-mm mesh screen (Cyclone Mill, UDY Mfg., Fort Collins, CO). Each sample was scanned by near-infrared reflectance spectroscopy. A FOSS 5000 scanning monochromator (FOSS, Danmark) was used for the collection of the reflectance measurements (log 1/R) between 1100 and 2500 nm, recorded at 2-nm intervals. The Coefficients of determination (R^2^) were 0.9589 for CP, 0.9573 for RUP, 0.8638 for K, 0.8243 for Ca, 0.7348 for Mg, and 0.6452 for P.

### Genotyping

Eighty-five polymorphic SSRs were used for genotyping the alfalfa panel [29, 30]. Genotyping analysis have been described by Qiang et al. [31]. The genotype data of 336 alfalfa genotypes were deposited in Additional file 4.

### Data analysis

The analysis of variance (ANOVA) of all phenotypic data was conducted using the general linear model in the SAS 8.02 [32]. The broad-sense heritability was calculated as *H* = σ_g_^2^/ (σ_g_^2^ + σ_e_^2^/n), where σ_g_^2^ is the genotypic variance, σ_e_^2^ is the environmental variance, and n is the number of the replications.

Genotypic data were filtered using a 5% cutoff value for minor allele frequency using the Tassel v2.1 software [33]. The association analysis was conducted using Tassel v2.1 based on three models, namely general linear model (GLM), Q and the compressed mixed linear model (MLM, Q + K) [34]. The association analysis was performed separately for each year’s phenotypic data, while the comparison of the three models was done using the mean values of 3 years’ data. The Bayesian model-based program STRUCTURE 2.2 [35] was used to infer the population structure (Q) which was described in the previous report [31]. The kinship matrix (K) was calculated using SPAGeDi software [36]. The markers were identified as significantly associated with traits at a significant level of *P* < 0.005.

## Additional files

Additional file 1: List of associated SSR alleles of six studied traits in 3 years. (XLSX 109 kb)

Additional file 2: The information about Mean monthly temperature, and precipitation at the experimental location in 3 years. (TIFF 1750 kb)

Additional file 3: Accession no. origin, improvement status, cultivar name, and No. of genotypes sampled of 336 alfalfa genotypes. (XLSX 12 kb)

Additional file 4: The genotype data of 336 alfalfa genotypes. (XLSX 497 kb)

## Abbreviations

ANOVA: Analysis of variance
CP: Crude protein
GLM: General linear model
GWAS: Genome-wide association study
MTAs: Marker-trait associations
QTLs: Quantitative trait loci
RUP: Rumen un-degraded protein
SSR: Simple sequence repeat

## References

[1] Castillo AR, Kebreab E, Beever DE, Barbi JH, Sutton JD, Kirby HC, et al. The effect of protein supplementation on nitrogen utilization in lactating dairy cows fed grass silage diets. J Anim Sci. 2001;79(1):247–53. 10.2527/2001.791247x 11204707

[2] Holt MS, Neal K, Eun JS, Young AJ, Hall JO, Nestor KE (2013). Corn silage hybrid type and quality of alfalfa hay affect dietary nitrogen utilization by early lactating dairy cows1. J Dairy Sci.

[3] Jarrett JP, Taylor MS, Nennich TD, Knowlton KF, Harrison J, Block E (2012). Effect of dietary calcium and stage of lactation on potassium balance in lactating Holstein cows through 20 weeks of lactation. The Professional Anim Scientist.

[4] Harrison J, White R, Kincaid R, Block E, Jenkins T, St-Pierre N (2012). Effectiveness of potassium carbonate sesquihydrate to increase dietary cation-anion difference in early lactation cows. J Dairy Sci.

[5] Goff JP, Brummer EC, Henning SJ, Doorenbos RK, Horst RL. Effect of application of ammonium chloride and calcium chloride on alfalfa cation-anion content and yield. J Dairy Sci. 90(11):5159–64. 10.3168/jds.2007-0070 17954756

[6] Garcia-Oliveira AL, Tan L, Fu Y, Sun C (2009). Genetic identification of quantitative trait loci for contents of mineral nutrients in rice grain. J Integr Plant Biol.

[7] Zhang M, Pinson SR, Tarpley L, Huang XY, Lahner B, Yakubova E, et al. Mapping and validation of quantitative trait loci associated with concentrations of 16 elements in unmilled rice grain. Theor Appl Genet. 2014;127(1):137–65. 10.1007/s00122-013-2207-5 PMC4544570 24231918

[8] Huang XQ, Cloutier S, Lycar L, Radovanovic N, Humphreys DG, Noll JS, et al. Molecular detection of QTLs for agronomic and quality traits in a doubled haploid population derived from two Canadian wheats (*Triticum aestivum* L.). Theor Appl Genet. 2006;113(4):753–66. 10.1007/s00122-006-0346-7 16838135

[9] Su J, Xiao Y, Li M, Liu Q, Li B, Tong Y, et al. Mapping QTLs for phosphorus-deficiency tolerance at wheat seedling stage. Plant Soil. 281(1):25–36.

[10] Yang M, Ding G, Shi L, Xu F, Meng J (2010). Detection of QTL for phosphorus efficiency at vegetative stage in *Brassica napus*. Plant Soil.

[11] Jin TT, Zhou JF, Chen JT, Zhu LY, Zhao YF, Huang YQ (2013). The genetic architecture of zinc and iron content in maize grains as revealed by QTL mapping and meta-analysis. Breed Sci.

[12] Qin H, Cai Y, Liu Z, Wang G, Wang J, Guo Y, et al. Identification of QTL for zinc and iron concentration in maize kernel and cob. Euphytica. 2012;187(3):345–58.

[13] Zhu CS, Gore M, Buckler ES, Yu JM (2008). Status and prospects of association mapping in plants. Plant Genome-Us.

[14] Font IFC, Velasco L, Socias ICR, Fernandez IMA (2015). Association mapping for kernel phytosterol content in almond. Front Plant Sci.

[15] Li YH, Reif JC, Ma YS, Hong HL, Liu ZX, Chang RZ, et al. Targeted association mapping demonstrating the complex molecular genetics of fatty acid formation in soybean. BMC Genomics. 2015;16:841. 10.1186/s12864-015-2049-4 PMC4619020 26494482

[16] Lu Q, Zhang M, Niu X, Wang S, Xu Q, Feng Y, et al. Genetic variation and association mapping for 12 agronomic traits in indica rice. BMC Genomics. 2015;16(1):1067. 10.1186/s12864-015-2245-2 PMC4681178 26673149

[17] Tadesse W, Ogbonnaya FC, Jighly A, Sanchez-Garcia M, Sohail Q, Rajaram S, et al. Genome-wide association mapping of yield and grain quality traits in winter wheat genotypes. PLoS One. 2015;10(10):e0141339. 10.1371/journal.pone.0141339 PMC4619745 26496075

[18] Zhang J, Zhao J, Xu Y, Liang J, Chang P, Yan F, et al. Genome-wide association mapping for tomato volatiles positively contributing to tomato flavor. Front Plant Sci. 2015;6:1042. 10.3389/fpls.2015.01042 PMC4661238 26640472

[19] Huang Y, Sun C, Min J, Chen Y, Tong C, Bao J (2015). Association mapping of quantitative trait loci for mineral element contents in whole grain rice (*Oryza sativa* L.). J Agric Food Chem.

[20] Nawaz Z, Kakar KU, Li XB, Li S, Zhang B, Shou HX, et al. Genome-wide Association mapping of quantitative trait loci (QTLs) for contents of eight elements in brown rice (*Oryza sativa* L.). J Agric Food Chem. 2015;63(36):8008–16. 10.1021/acs.jafc.5b01191 26317332

[21] Yu J, Buckler ES (2006). Genetic association mapping and genome organization of maize. Curr Opin Biotechnol.

[22] Li X, Wei Y, Moore KJ, Michaud R, Viands DR, Hansen JL (2011). Association mapping of biomass yield and stem composition in a tetraploid alfalfa breeding population. Plant Genome.

[23] Yu LX, Liu X, Boge W, Liu XP (2016). Genome-wide association study identifies loci for salt tolerance during germination in autotetraploid alfalfa (*Medicago sativa* L.) using genotyping-by-sequencing. Front. Plant Sci.

[24] Sakiroglu M, Brummer EC. Identification of loci controlling forage yield and nutritive value in diploid alfalfa using GBS-GWAS. Theor Appl Genet. 2016; doi:10.1007/s00122-016-2782-3. 10.1007/s00122-016-2782-3 27662844

[25] Liu Y, Wang L, Mao S, Liu K, Lu Y, Wang J, et al. Genome-wide association study of 29 morphological traits in *Aegilops tauschii*. Sci Rep-UK. 2015;5:15562. 10.1038/srep15562 PMC4622089 26503608

[26] Sun X, Du Z, Ren J, Amombo E, Hu T, Fu J (2015). Association of SSR markers with functional traits from heat stress in diverse tall fescue accessions. BMC Plant Biol.

[27] Du J, Zeng D, Wang B, Qian Q, Zheng S, Ling HQ (2013). Environmental effects on mineral accumulation in rice grains and identification of ecological specific QTLs. Environ Geochem Hlth.

[28] Wang Z, Qiang HP, Zhao HM, Xu RX, Zhang ZL, Gao HW, et al. Association mapping for fiber-related traits and digestibility in alfalfa. Front Plant Sci. 2016;7:331. 10.3389/fpls.2016.00331 PMC4797558 27047512

[29] Eujayl I, Sledge MK, Wang L, May GD, Chekhovskiy K, Zwonitzer JC, et al. *Medicago truncatula* EST-SSRs reveal cross-species genetic markers for *Medicago* spp. Theor Appl Genet. 2004;108(3):414–22. 10.1007/s00122-003-1450-6 13679975

[30] Robins JG, Luth D, Campbell IA, Bauchan GR, He CL, Viands DR, et al. Genetic mapping of biomass production in tetraploid alfalfa. Crop Sci. 2007;47(1):1–10.

[31] Qiang HP, Chen ZH, Zhang ZL, Wang XM, Gao HW, Wang Z. Molecular diversity and population structure of a worldwide collection of cultivated tetraploid alfalfa (*Medicago sativa subsp sativa* L.) germplasm as revealed by microsatellite markers. PloS One. 2015;10(4):e0124592. 10.1371/journal.pone.0124592 PMC4406709 25901573

[32] SAS Institute Inc. SAS/STAT 8.2 User's guide. Cary: SAS Institute Inc; 1999.

[33] Bradbury PJ, Zhang Z, Kroon DE, Casstevens TM, Ramdoss Y, Buckler ES (2007). TASSEL: software for association mapping of complex traits in diverse samples. Bioinformatics.

[34] Yu JM, Pressoir G, Briggs WH, Bi IV, Yamasaki M, Doebley JF, et al. A unified mixed-model method for association mapping that accounts for multiple levels of relatedness. Nat Genet. 2006;38(2):203–8. 10.1038/ng1702 16380716

[35] Pritchard JK, Stephens M, Donnelly P (2000). Inference of population structure using multilocus genotype data. Genetics.

[36] Hardy OJ, Vekemans X (2002). SPAGeDi: A versatile computer program to analyses spatial genetic structure at the individual or population levels. Mol Ecol Notes.

