# Supplementary figures and images for: Identification of genetic loci associated with crude protein and mineral concentrations in alfalfa (Medicago sativa) using association mapping
Source: BMC Plant Biol. 2017 Jun 6;17:97. doi: 10.1186/s12870-017-1047-x (PMC5460482; doi:10.1186/s12870-017-1047-x)

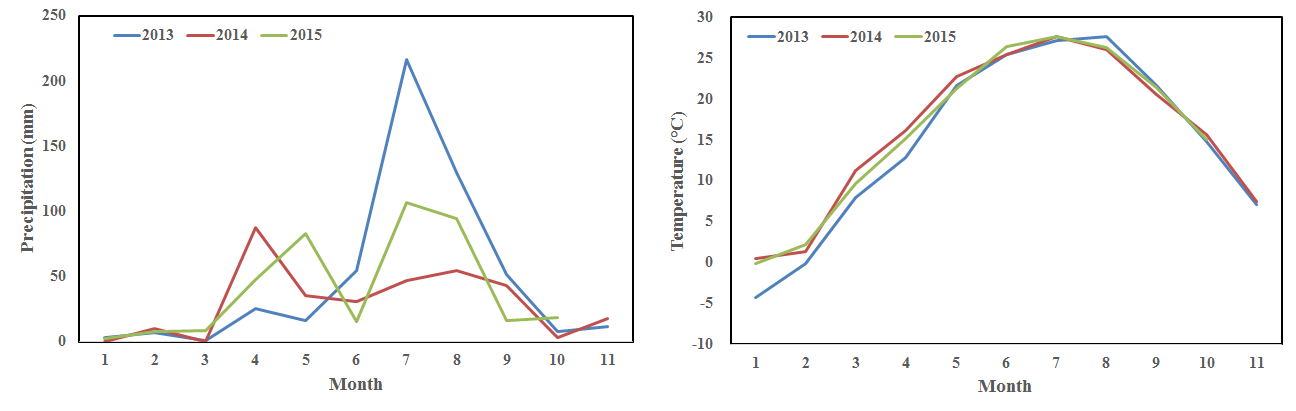

Supplement: Supplementary file 2 — The information about Mean monthly temperature, and precipitation at the experimental location in 3 years. (TIFF 1750 kb) [file 12870_2017_1047_MOESM2_ESM.tif]
